# PPAR**γ** Ligands Regulate Noncontractile and Contractile Functions of Airway Smooth Muscle: Implications for Asthma Therapy

**DOI:** 10.1155/2012/809164

**Published:** 2012-08-16

**Authors:** Chantal Donovan, Xiahui Tan, Jane Elizabeth Bourke

**Affiliations:** Department of Pharmacology, University of Melbourne, Parkville, VIC 3010, Australia

## Abstract

In asthma, the increase in airway smooth muscle (ASM) can contribute to inflammation, airway wall remodeling and airway hyperresponsiveness (AHR). Targetting peroxisome proliferator-activated receptor **γ** (PPAR**γ**), a receptor upregulated in ASM in asthmatic airways, may provide a novel approach to regulate these contributions. This review summarises experimental evidence that PPAR**γ** ligands, such as rosiglitazone (RGZ) and pioglitazone (PGZ), inhibit proliferation and inflammatory cytokine production from ASM *in vitro*. In addition, inhaled administration of these ligands reduces inflammatory cell infiltration and airway remodelling in mouse models of allergen-induced airways disease. PPAR**γ** ligands can also regulate ASM contractility, with acute treatment eliciting relaxation of mouse trachea *in vitro *through a PPAR**γ**-independent mechanism. Chronic treatment can protect against the loss of bronchodilator sensitivity to **β**_2_-adrenoceptor agonists and inhibit the development of AHR associated with exposure to nicotine *in utero *or following allergen challenge. Of particular interest, a small clinical trial has shown that oral RGZ treatment improves lung function in smokers with asthma, a group that is generally unresponsive to conventional steroid treatment. These combined findings support further investigation of the potential for PPAR**γ** agonists to target the noncontractile and contractile functions of ASM to improve outcomes for patients with poorly controlled asthma.

## 1. Introduction 

Asthma is a chronic inflammatory lung disease affecting over 300 million people worldwide, with 250,000 deaths per year attributed to the disease [1]. Asthma is characterized by inflammation, airway wall remodeling, and airway hyperresponsiveness (AHR), whereby airways are more sensitive to a variety of stimuli and subsequently contract too easily and too much [2].

**Figure 1:**
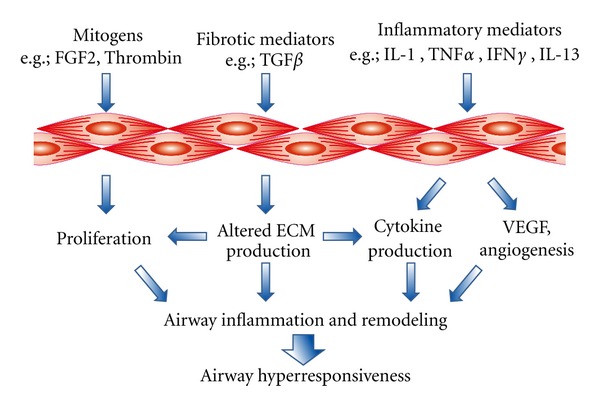
Potential targets for the regulation of noncontractile (proliferative and synthetic) and contractile functions of airway smooth muscle contributing to airway hyperresponsiveness.

A major feature of airway remodeling in asthma is an increase in airway smooth muscle (ASM) mass. This thickened ASM layer can act as both a source and target of inflammatory cytokines and extracellular matrix (ECM) proteins, contributing to persistent inflammation and increased airway narrowing. Proliferative, synthetic, and contractile functions of ASM can therefore play distinct roles in both the pathogenesis of asthma and perpetuation of disease symptoms (Figure 1) [3, 4].

In current asthma therapy, inhaled *β*_2_-adrenoceptor agonists are used to reverse ASM contraction while the frequency and severity of asthma attacks can be reduced by combined therapy with *β*_2_-adrenoceptor agonists and glucocorticoids (GCS). However, a significant proportion of patients have poorly controlled symptoms, with variable responses to *β*_2_-adrenoceptor agonists and persistent AHR despite optimal anti-inflammatory treatment. Cigarette smoking in asthma patients also contributes to increased severity of symptoms, with an impaired response to both inhaled and oral corticosteroids [5].

This resistance to therapy is likely to be associated with significant structural changes to the airways, including ASM accumulation, fibrosis, and increased vascularity. These changes have been mechanistically associated with disease severity and accelerated lung function decline [6] but may be difficult to reverse in established asthma. In this context therefore, it is crucial to identify alternative drugs that inhibit the development of AHR, as well as the contribution of ASM to inflammation and remodeling to limit contraction or directly enhance the relaxation of the increased ASM in the airways [7, 8].

A potential novel approach to regulate ASM function in asthma is to target peroxisome proliferator-activated receptor *γ* (PPAR*γ*). It has been suggested that downregulation of PPAR*γ* signaling may be a contributing factor to the development of AHR in asthma following *in utero* nicotine exposure [9], while the expression of PPAR*γ* in ASM is upregulated in the airways of asthmatic patients [10]. 

This paper provides a brief overview of PPAR*γ* pharmacology and describes the contribution of ASM to inflammation, remodeling, and hyperresponsiveness in asthma. Its major focus is to outline the increasing experimental and clinical evidence that PPAR*γ* ligands can regulate ASM function, through both PPAR*γ*-dependent and PPAR*γ*-independent mechanisms. 

## 2. PPAR*γ* Structure and Ligands

PPAR*γ* is a member of the nuclear hormone receptor (NHR) family of ligand-activated transcription factors, which also includes glucocorticoid receptors (GRs) and thyroid hormone receptors. PPAR*γ* is one of three PPAR isoforms designated PPAR*α* (NR1C1), PPAR*β* (PPAR*δ*, NR1C2), and PPAR*γ* (NR1C3).

Like other NHR, PPAR*γ* possesses a multidomain structure. This includes a DNA binding domain (DBD) containing two zinc finger motifs that recognise specific PPAR response elements (PPREs) sequences. These PPREs consist of hexameric direct repeats of AGGTCA recognition sequences separated by one or two random nucleotide. The DBD is linked via a hinge region to the large ligand binding domain (LBD) that occupies the C-terminal half of the receptor and an activator function (AF)-1 domain is present at the N-terminus [11–13].

PPAR*γ* possesses an unusually large T-shaped ligand-binding pocket that enables interaction with a structurally diverse library of ligands [13]. The most widely studied synthetic agonists are the thiazolidinedione class of drugs, rosiglitazone (RGZ, BRL 49653), pioglitazone (PGZ), troglitazone (TGZ), and ciglitazone (CGZ). RGZ binds the receptor with high affinity (K_d_ 43 nM), whereas PGZ and CGZ are less potent [14]. Alternative nonglitazone agonists include GW262570 [15] and novel triterpenoid compounds derived from oleanic acid such as 2-cyano-3,12-dioxoolean-1,9-dien-28-oic acid (CDDO) [16]. Despite binding affinities in the nM range, most biological effects of these synthetic PPAR*γ* agonists have been observed at *μ*M concentrations.

Potential natural ligands for PPAR*γ* also show marked structural diversity and include prostaglandin D_2_ (PGD_2_) and its metabolites PGJ_2_ and 15-deoxy-^Δ12,14^-prostaglandin-J_2_ (15dPGJ_2_) [17]. 15dPGJ_2_ in particular has been widely used for comparisons with glitazones in experimental settings [18]. However, these agonists, and other putative PPAR*γ* ligands such as the oxidised lipids 9- and 13-hydroxyoctadecadienoic acid (HODE) and 12- and 15-hydroxyeicosatetraenoic acid (HETE) [19], have multiple additional sites of action, suggesting that demonstrating their PPAR*γ*-dependent actions is likely to be particularly challenging.

## 3. Mechanisms of Action of PPAR*γ* and ****Its Ligands

### 3.1. PPAR*γ* Activation

Cytosolic PPAR*γ* exists as a monomer, with both the LBD and AF-1 domain regulating interactions with coactivators and corepressors that control activation of the receptor [11–13]. PPAR*γ* does not form homodimers, but can associate with multiple partners to form heterodimers. Its most preferential binding partner is the retinoid X receptor (RXR), with 9-cis retinoic acid acting as its natural ligand [20]. Translocation of the ligand-activated PPAR*γ*-RXR complex to the nucleus and binding to PPRE in the promoter region of target genes can result in either the upregulation or inhibition of gene expression. Multiple PPAR*γ*-responsive genes involved in diverse cellular processes including adipogenesis, insulin sensitivity, and inflammation have been identified [21, 22].

Alternatively, PPAR*γ* can cause the transrepression of transcriptional factors such as NF*κ*B, CAAT/enhancer binding protein (C/EBP), signal transducers and activators of transcription (STAT), or activator protein- (AP-) 1. This transrepression may occur either by direct binding to the transcription factors, by sequestration of shared coactivators of these factors or by Small Ubiquitin-like Modifier (SUMO) ylation of PPAR*γ* and subsequent PPAR*γ* binding to a corepressor complex [23]. These actions also have the potential to inhibit inflammatory responses in the lung. 

Given the marked differences between the reported PPAR*γ* binding affinities of the glitazones and the concentrations required to elicit their cellular effects, multiple approaches are required to support claims for PPAR*γ*-dependency. These include confirming PPAR*γ* expression in cells of interest and the use of pharmacological antagonists, with GW9662 being the most commonly used. GW9662 irreversibly inhibits PPAR*γ* by covalently binding to Cys^285^ in the ligand binding pocket and prevents heterodimerisation with RXR as well as interactions with coactivator and corepressor molecules [24]. GW9662 has been used to confirm the PPAR*γ*-dependence of known PPAR*γ* ligands both *in vitro* [18] and *in vivo* [25]. Additional molecular techniques such as the use of dominant-negative constructs or adenoviral PPAR*γ* (AdPPAR*γ*) have been employed to implicate PPAR*γ* in the regulation of cellular responses both *in vitro *and* in vivo* [26, 27].

### 3.2. PPAR*γ*-Independent Mechanisms

The mechanistic complexity underlying responses to PPAR*γ* ligands occurring via PPAR*γ* activation is further complicated by evidence of PPAR*γ*-independent pathways.This may involve PPAR*γ* ligands binding directly to alternative receptors, regulating transcription factor activity, or altering signalling through enzymes or ion channels to mediate their cellular responses.

Glitazones have been shown to activate free fatty acid receptors (FFA1, also known as GPR40) causing phosphorylation of the ERK1/2 mitogen-activated protein (MAP) kinases [28]. RGZ and CGZ can also bind directly to GR independently of PPAR*γ*, evidenced by their stimulation of GR nuclear translocation in a PPAR*γ*-deficient cell line [29], and defining a potential alternative anti-inflammatory mechanism for these ligands.

In addition, 15dPGJ_2_ has been demonstrated to directly inhibit the activity of the enzyme I*κ*B kinase, thereby reducing the phosphorylation of I*κ*B and its subsequent dissociation from the proinflammatory transcription factor NF*κ*B [30, 31]. Actions of PPAR*γ* ligands may also be mediated by increasing PGE_2_ levels, subsequent to inhibition of its metabolism via 15-hydroxyprostaglandin dehydrogenase [32].

Both 15dPGJ_2_ and CDDO have been shown to induce heme-oxidase by PPAR*γ*-independent, glutathione-dependent mechanism, although this antioxidant action was restricted to PPAR*γ* ligands possessing an electrophilic centre [33]. Additional evidence of nongenomic, rapid regulation of enzyme activity by PPAR*γ* ligands includes activation of mitogen-associated protein kinase (MAPK), phosphoinositide-3-kinase (PI3K), and adenosine monophosphate-activated protein kinase (AMPK) pathways [34–36] with implications for regulation of cell proliferation and inflammatory cytokine production.

Some of these nongenomic effects may be mediated through altered calcium signalling. TGZ and PGZ have been shown to mobilize calcium from intracellular stores [37, 38]. Although RGZ rapidly inhibited the activity of sarco/endoplasmic reticulum Ca^2+^ ATPase (SERCA)-2b [39], chronic treatment with PGZ has been shown to increase SERCA activity via a PPAR*γ*-dependent mechanism [40], suggesting that regulation of calcium homeostasis by PPAR*γ* ligands is likely to depend on temporal and cellular contexts.

These diverse PPAR*γ*-dependent and PPAR*γ*-independent actions define numerous mechanisms whereby PPAR*γ* ligands could regulate the altered proliferative, secretory and contractile functions of ASM contributing to asthma.

## 4. PPAR*γ* Is Increased in Airway Smooth Muscle in Asthma

Although PPAR*γ* was originally characterised as a regulator of adipocyte differentiation, this receptor is also widely expressed in the lung, in both inflammatory and structural cells implicated in asthma pathophysiology [41]. 

Regulation of PPAR*γ* expression can occur in response to *in vitro* exposure to inflammatory cytokines, with acute upregulation occurring in response to interleukin-4 (IL-4) in airway epithelial cells and macrophages [42, 43] during macrophage differentiation and activation [44, 45] or following antigen exposure in sensitised mast cells [46]. 

PPAR*γ* may provide a target to overcome chronic inflammation and increased airway reactivity *in vivo*. Higher levels of PPAR*γ* were evident in total lung extracts from mouse models of established allergen-induced inflammation [27, 47] and could be localised to ASM and epithelium, mast cells, and some inflammatory cells [25]. In contrast, perinatal exposure to nicotine appears to decrease PPAR*γ* expression and signaling, with increased alveolar interstitial fibroblast-to-myofibroblast differentiation contributing to the development of an asthma-like phenotype in newborn rats [48, 49].

In human airway biopsies, expression levels of PPAR*γ* in ASM, epithelium, and mucosal eosinophils and macrophages were elevated in asthmatic patients compared with controls [10]. In the same study, asthmatics treated with GCS had lower levels of PPAR*γ* expression compared with untreated asthmatics. Although ASM from asthmatics had lower PPAR*γ* levels compared to healthy controls *in vitro*, this was reversed in the presence of a mitogenic stimulus [50]. These results suggest that increased PPAR*γ* expression observed *in situ* may be a product of the inflammatory and mitogenic pathways and may also be sensitive to steroid therapy. 

These combined findings suggest that PPAR*γ* expression is increased in response to acute or chronic inflammation in multiple cell types including ASM, and that PPAR*γ* could be targeted to limit inflammation, airway remodeling, and increased ASM contraction in asthma.

## 5. *In Vitro* Regulation of ASM Function by**** PPAR*γ* Ligands

Because of the capacity of ASM to perpetuate airway inflammation, orchestrate airway wall remodelling, and modulate airway tone, it has been suggested that targeting ASM is critical for effective asthma treatment [4, 7, 8]. Accumulating *in vitro* evidence now supports the efficacy of PPAR*γ* ligands in the regulation of ASM cytokine production, proliferation, and contraction, while their direct effects on the potential contribution of ASM to fibrosis and angiogenesis have yet to be confirmed. 

### 5.1. Regulation of ASM Inflammatory Cytokine Production

In response to inflammatory stimuli, ASM can secrete various cytokines and chemokines contributing to the pathophysiology of asthma [51–53]. These mediators include factors such as granulocyte/macrophage-colony-stimulating factor (GM-CSF) [54], granulocyte-colony stimulating factor (G-CSF) [55], eotaxin [56], regulated on activation, normal T cells expressed and secreted (RANTES) [57], IL-6 [58], monocyte chemotactic protein-1 (MCP-1) [59], and vascular endothelial growth factor (VEGF) [60]. Thus, inhibition of ASM cytokine production has the potential to regulate inflammatory cell growth, survival, and recruitment as well as the autocrine influence of these cytokines to induce ASM proliferation and hyperresponsiveness in the inflamed airways in asthma [4, 61].

The anti-inflammatory efficacy of PPAR*γ* ligands has been established in human-cultured ASM cells, with various glitazones inhibiting the release of multiple cytokines irrespective of stimulus used. IL-1*β*-induced increases in GM-CSF and G-CSF release were both attenuated by CGZ and to a lesser extent by 15dPGJ_2_ [55]. Although dexamethasone also completely abolished the increase in GM-CSF release, G-CSF induction was only partially inhibited, suggesting that PPAR*γ* ligands may target steroid-resistant pathways in ASM [55]. Induction of eotaxin and MCP-1 by tumour necrosis factor *α* (TNF*α*) was also inhibited by PPAR*γ* agonists, and the expression of these chemokines was further decreased when 15dPGJ_2_ and TGZ were used in the presence of GCS and/or a long-acting *β*-adrenoceptor agonists [62], supporting potential benefit when these agents are used in combination.

In a more recent study, TGZ inhibited IL-1*β*-induced release of IL-6 and VEGF, TNF*α*-induced release of eotaxin and RANTES, and IL-4-induced release of eotaxin, while RGZ also inhibited TNF*α*-stimulated release of RANTES. These anti-inflammatory effects were not prevented by the PPAR*γ* antagonist GW 9662 or by PPAR*γ* knockdown using short hairpin RNA [63].

Additional PPAR*γ*-independent mechanisms have been considered. Although PPAR*γ* ligands each caused the activation of AMPK, their effects on cytokine release were not prevented by AMPK inhibitors [63]. Since CGZ increased the IL-1*β*-induced expression of COX-2 [22], this potentially proinflammatory effect could also contribute to increased PGE_2_ levels to provide negative feedback to inhibit cytokine release [64]. However, induction of PGE_2_ synthesis was not a requirement for the anti-inflammatory effect of PPAR*γ* ligands, since CGZ reduced GM-CSF and G-CSF in the presence of indomethacin [55]. Inhibition of NF*κ*B has also been excluded, since CGZ did not regulate NF*κ*B (p65) nuclear translocation in the absence or presence of IL-1*β* [22].

The qualitative importance of ASM-derived cytokines remains to be clearly established *in vivo* and in asthmatic subjects. Nevertheless, evidence of the diverse anti-inflammatory actions of PPAR*γ* ligands in ASM, consistent with their reported actions in other cell types [41], supports their therapeutic potential for the treatment of asthma. 

### 5.2. Regulation of ASM Proliferation

A key feature in airway remodeling in asthma is the increased ASM layer associated with increases in both size (hypertrophy) and number (hyperplasia, migration) of myocytes [65] with ASM cell migration also playing a potential role. To assess the potential efficacy of antiremodeling agents, ASM proliferation can be induced *in vitro *in response to the cocktail of mitogens present in serum, and to specific stimuli such as thrombin or fibroblast growth factor 2 (FGF2), known to be increased in the asthmatic airway [66, 67].

PPAR*γ* ligands have now been shown to inhibit proliferation of human ASM in culture. The increase in [^3^H]-thymidine incorporation in response to serum was completely abolished by both CGZ and 15dPGJ_2_ [55], while RGZ and 15dPGJ_2_ significantly attenuated both FGF2 and thrombin-stimulated increase in ASM cell numbers [18], demonstrating that the antiproliferative effects are mitogen-independent. Unlike GCS, inhibition of proliferation was not associated with reduced cyclin D1 levels [18, 68]. Responses were mediated by both PPAR*γ*-dependent and PPAR*γ*-independent mechanisms, as the PPAR*γ* antagonist GW9662 inhibited the antiproliferative effects of RGZ but not 15dPGJ_2_ [18], with cell cycle analysis suggesting that neither mediated ASM apoptosis [18]. Although CGZ and 15dPGJ_2_ had previously been reported to cause nuclear condensation, a characteristic morphological change associated with apoptosis [55], this single finding was not consistent with the known resistance of ASM to apoptosis [69].

Cultured ASM derived from asthmatic patients has been shown to proliferate faster than cells from nonasthmatic patients [70]. Since GCS can only inhibit the *in vitro* proliferation of ASM from subjects without asthma [68, 71], alternative therapeutic approaches are required to target this steroid-resistant mitogenic response. In a recent study, the effects of CGZ were assessed in cells from nonasthmatic and asthmatic patients cellular proliferation in response to serum by measuring bromodeoxyuridine uptake [50]. Further studies are required to explain why CGZ failed to inhibit serum-induced proliferation in either group [50], since this finding contradicts the previously reported antiproliferative effects of both CGZ [55] and RGZ [18]. CGZ did upregulate PPAR*γ* expression in ASM cells derived from both asthmatic and nonasthmatic subjects, and in ASM from asthmatics in the presence of serum [50], however the functional significance of these changes and their potential impact on ASM in remodeled airways remain to be determined.

### 5.3. Regulation of Extracellular Matrix Production and Turnover

Airway remodeling in asthma is also characterized by alterations in the amount and composition of ECM proteins, including increases in collagen I and fibronectin deposition [72]. Subepithelial fibrosis is associated with increased transforming growth factor *β* (TGF*β*), with this profibrotic cytokine present at relatively higher levels in BAL fluid from asthmatic subjects compared to healthy subjects [73]. Although fibroblasts are considered the major resident cells contributing to the increased collagen deposition in the asthmatic airway, ASM is also known to produce ECM proteins and to regulate their turnover by secreting matrix modifying enzymes.

In this context, it is important to consider that the ECM exists not only as a structural scaffold in the airways but as a partner in bidirectional interactions with ASM, influencing proliferation and cytokine release as well as contractility [74]. Since *in vitro* secretion of collagen and fibronectin from ASM derived from asthmatic patients is increased by GCS, and TGF*β*-induced ECM protein synthesis is unaffected by GCS, this aspect of remodeling appears to be resistant to steroids [75] and alternative strategies to minimize the impact of the altered ECM on ASM function need to be identified.

Confirmation of the ability of PPAR*γ* ligands to inhibit TGF*β*-induced collagen synthesis from ASM would suggest that these agents have the capacity oppose proasthmatic changes associated with increased ASM-ECM interactions. To date, the effects of PPAR*γ* ligands have only been assessed in human lung fibroblasts which express PPAR*γ*, and respond to TGF*β* treatment by differentiating into myofibroblasts expressing *α* smooth muscle actin (*α*SMA), and increasing their synthesis of fibrillar collagen I [26]. Both differentiation and collagen I secretion were abrogated by treatment with RGZ, CGZ, or 15dPGJ_2_. These antifibrotic effects of the PPAR*γ* ligands were shown to be at least partially mediated by PPAR*γ* receptor activation as inhibition was attenuated by transfection of TGF*β*-treated fibroblasts with a dominant negative PPAR*γ* receptor [26]. Similar antifibrotic properties have also been described for RGZ and CGZ in the regulation of epithelial-mesenchymal transition in alveolar epithelial cells [76].

An alternative way to regulate ASM-ECM interactions would be by regulating the activity of matrix metalloproteinases (MMPs) and their tissue inhibitors (TIMPs). Activation of PPAR*γ* by RGZ or PGZ in human bronchial epithelial cells reduced TNF*α*-induced MMP-9 gelatinolytic activity via inhibition of NF-*κ*B, but did not alter the expression of its endogenous inhibitor TIMP-1 [77]. These results suggest that limiting the expression of MMP-9 by PPAR*γ* activation might have therapeutic potential in the treatment of chronic inflammatory diseases of the respiratory system. However, the effects of PPAR*γ* ligands on ASM-derived MMPs and TIMPs in the asthma context have yet to be directly assessed.

### 5.4. Regulation of Angiogenesis

Significant increases in the number and size of blood vessels supplying the remodeled airway wall are seen in asthma [6, 78]. This expanded vascular compartment is likely to contribute to asthma symptoms through tissue swelling and amplification of inflammatory cell trafficking [79]. ASM has the potential to promote angiogenesis as cultured ASM has been shown to constitutively release factors such as VEGF, which can be further increased in response to inflammatory mediators such as IL-1*β*, TNF*α*, and TGF*β* [80]. Of note, these proangiogenic responses have recently been shown to be further elevated in ASM from asthmatics [81].

Studies examining the effects of PPAR*γ* ligands on this aspect of remodelling are lacking; however, conflicting reports show that the generation of VEGF from vascular smooth muscle cells is increased by CGZ and PGJ_2_ [82, 83], while TGZ has been shown to inhibit VEGF-induced angiogenic signaling in endothelial cells [84]. Further investigations are required to explore the potential of PPAR*γ* ligands to regulate the contribution of ASM to angiogenesis.

### 5.5. Regulation of ASM Contraction

The increased contractile response of asthmatic airways which defines AHR is likely to be due to multiple factors (recently reviewed in [4]), including the presence of higher levels of contractile mediators and reduced levels of relaxant mediators. Critically, the increased ASM bulk displays alterations in contractile protein expression that favour contraction [85, 86]. In this context, it is of interest that RGZ and other PPAR*γ* ligands can inhibit the increase in *α*-smooth muscle actin and calponin associated with both epithelial-mesenchymal transition of alveolar epithelial cells [76] and alveolar interstitial fibroblast-to-myofibroblast differentiation [87].

Increased excitation/contraction coupling may also occur through disruption of calcium homeostasis [88]. Indeed, increased contraction of ASM cells from asthmatic patients has been associated with downregulation in their expression and function of SERCA2 [89]. PPAR*γ* ligands have recently been reported to increase SERCA expression and activity in pancreatic islet cells and platelets [40, 90], with PGZ inhibiting cytokine-induced increases in intracellular calcium by facilitating its reuptake into the SR [40]. In ASM, calcium plays a key role not only in enhancing ASM contractile function, but also in promoting cell proliferation, migration and the secretion of proinflammatory cytokines and chemokines [88]. It will therefore be of particular interest to determine if acute or chronic treatment with PPAR*γ* ligands can also restore SERCA levels and activity in ASM to inhibit the diverse proasthmatic functions that could be driven by elevated intracellular calcium.

There is now evidence that acute treatment with PPAR*γ* ligands may exert direct effects on ASM contractility. In a single study, RGZ has been reported to cause relaxation of mouse tracheal preparations precontracted with carbachol [91]. Since this response was evident within minutes and required *μ*M concentrations, it was likely to be occurring independently of PPAR*γ* activation. Relaxation to RGZ in the static organ bath setting was indomethacin-sensitive and was attributed to accumulation of the dilator prostanoid PGE_2_ through inhibition of its breakdown rather than an increase in PGE_2_ synthesis. This interpretation is consistent with the previously reported finding that RGZ can inhibit its metabolism by 15-hydroxyprostaglandin dehydrogenase [32].

Further studies are required to explore acute dilator responses to RGZ and other PPAR*γ* ligands, to compare their efficacy with *β*_2_-adrenoceptor agonists in current clinical use for the relief of asthma symptoms and to test their actions in the disease context when ASM responsiveness is altered.

## 6. *In Vivo* Regulation of ASM Function by**** PPAR*γ* Ligands

### 6.1. PPAR*γ* Ligands Have Efficacy in Rodent Models of Allergic and Nicotine-Induced Airways Disease

The reported effects of PPAR*γ* ligands on ASM functions *in vitro*, namely inhibition of proliferation and production of cytokines from human ASM cells as well as regulation of contractile protein expression and direct relaxation intracheal preparations, has provided an impetus for considering their effects in animal models of airways disease, using perinatal exposure to maternal nicotine or chronic ovalbumin (OVA) challenge to trigger asthma-like changes in the airways.

It is well known that cigarette smoking during pregnancy increases the incidence and severity of childhood asthma, and has been associated with increased generation of contractile myofibroblasts in the developing lung (reviewed in [9]). A recent study of newborn rats following *in utero* nicotine exposure has revealed that increases in airway resistance under basal conditions and reactivity to acetylcholine both *in vivo* and* in vitro* are sensitive to RGZ treatment [48].

Chronic allergen challenge models mimic key features of asthma including inflammatory cell infiltration predominantly not only by eosinophils, but also by neutrophils and lymphocytes [92]. Increased inflammatory Th_2_ cytokines and chemotactic factors can also be detected in bronchoalveolar lavage (BAL) fluid [27, 93, 94]. Airway wall remodeling, with variable changes in goblet cell hyperplasia, thickening of the ASM layer and increased collagen deposition in the lamina propria is also evident [25, 95]. A common feature is the development of AHR to methacholine (MCh), demonstrated both with noninvasive, but now largely discredited [96] measurement of the heuristic variable enhanced pause (Penh) in conscious mice [25, 27, 94, 95] and by measurements of increased airways resistance using invasive plethysmography in anesthetized mice [47, 97, 98].

The efficacy of PPAR*γ* ligands in these OVA challenge models in mice has been confirmed in multiple studies, assessing the effect of chronic treatment with glitazones or other PPAR*γ* selective agonists such as GI 262570, administered either by inhaled, oral or intraperitoneal (i.p.) routes [25, 27, 47, 92–95, 97–100] or the response to transient overexpression of PPAR*γ* via adenoviral delivery (Table 1) [27, 47, 93]. However, since the overall changes in phenotype in these models can vary with the type of OVA used [101], the duration of the challenge protocol and the species of mice in which the model is applied [102], the reported effects of pharmacological intervention with different PPAR*γ* ligands administered by various routes must be considered and interpreted in context. Nevertheless, these models provide compelling evidence that PPAR*γ* ligands can regulate inflammatory cell infiltration, BAL cytokines, airway remodeling particularly ASM thickening and fibrosis, and altered reactivity to MCh.

### 6.2. Regulation of Airway Inflammation

Assessment of airway inflammation has consistently shown that RGZ or CGZ treatment attenuated the increase in total and eosinophil cell numbers in BAL fluid in OVA-treated C57Bl/6 or Balb/C mice in a PPAR*γ*-dependent manner (Table 1) [27, 93, 94]. Similar results were observed with GI 262570 administration to Balb/C mice, where eosinophil and lymphocytes, but not neutrophils, were reduced [92]. Although RGZ reduced eosinophilic airway inflammation when administered to Balb/C mice by oral gavage [99], it was ineffective in C57Bl/6 mice administered i.p. [98]. The reason for this discrepancy is therefore more likely to be due to differences in challenge protocols and the administration methods (route, dose, and duration) of different compounds, rather than the mouse strain used.

Regulation of cytokine production in the lung has also been assessed. OVA-induced increases in IL-4, IL-5, IL-13, eosinophil cationic protein (ECP), and eotaxin in lung tissue and BAL fluid were inhibited by administration of RGZ, PGZ, or by PPAR*γ* overexpression [27, 93]. Similar changes were seen with nebulized CGZ, although eotaxin levels were not affected [94], while oral CGZ has been shown to reduce IL-2, IL-4, and interferon *γ* (IFN*γ*) [100]. Since cytokine release from ASM is also inhibited by PPAR*γ* ligands* in vitro*, it is likely that the glitazones can reduce the contribution of ASM-derived cytokines to the levels measured in this *in vivo* setting.

Several potential mechanisms have been proposed to explain the anti-inflammatory effects of PPAR*γ* ligands in these models. Regulation of NF*κ*B has been considered since PPAR*γ* activation inhibits the function of the proinflammatory transcription factor *in vitro* [103, 104]. Treatment of OVA-sensitised mice with RGZ, PGZ, or AdPPAR*γ* also reduced the nuclear translocation of NF*κ*B in response to OVA, evidenced by inhibition of increases in NF*κ*B p65 protein in lung extracts [93], suggesting a direct action of PPAR*γ* ligands on NF*κ*B. Inhibition of NF*κ*B activity by PPAR*γ* agonists has also been associated with decreased IL-17 protein and mRNA expression. Since the effects of RGZ or PGZ could be abrogated by coadministration of rIL-17, this implicates a novel mechanism whereby PPAR*γ* agonists regulate NF*κ*B activity by reducing IL-17 to limit inflammation [97].

NF*κ*B-independent mechanisms are also likely to contribute to the anti-inflammatory effects of PPAR*γ* ligands [92]. Alternative mechanisms include PPAR*γ*-mediated inhibition of the increase in GATA-3 expression in response to OVA [94], reducing the local Th2 response elicited by this eosinophil-derived transcription factor. In addition, an increase in IL-10 in response to OVA, thought to occur as part of a negative feedback response to inhibit inflammation, could be further increased by RGZ, PGZ, or ad PPAR*γ* [47]. Increased IL-10 levels could explain the reported reductions in IL-4 and IL-5 as well as the inhibition of eosinophilia, since IL-10 has been shown to downregulate IL-4 and IL-5 expression by Th2 cells and reduce eosinophil survival. 

In a separate study, PPAR*γ* expression was increased in response to OVA challenge and further enhanced by the administration of the either PPAR*γ* agonists or AdPPAR*γ* [27]. This was associated with an upregulation of phosphatase and tensin homologue deleted on chromosome ten (PTEN) PTEN expression, correlating with decreased PI3K activity as measured by a reduction in the phosphorylation of Akt. These findings demonstrate a protective role of PPAR*γ* in the pathogenesis of the asthma phenotype through regulation of PTEN expression [27, 93].

### 6.3. Regulation of Airway Remodeling

In addition to their anti-inflammatory actions in these mouse models, PPAR*γ* ligands have also been shown to inhibit key aspects of airway remodeling, notably fibrosis, mucus production, and thickening of the ASM layer (Table 1).

Inhaled CGZ has been shown to reduce OVA-induced increases in both collagen deposition and basement membrane thickening [25]. This was associated with reduced levels of the profibrotic cytokine TGF*β* [25]. Although inhaled CGZ has also been shown to decrease mucus production, based on the intensity and area of epithelial staining [25], there were no detectable effects of i.p. RGZ on goblet cell number or other aspects of airway remodeling [98], suggesting that high local concentrations may be required.

Consistent with the reported antiproliferative effects of PPAR*γ* ligands on human ASM *in vitro* [18, 50, 55], intranasal administration of CGZ has been shown to reduce not only eosinophilic inflammation, but also to inhibit the thickening of the ASM layer following allergen challenge [95]. This effect appeared to be independent of regulation of TGF*β* or VEGF levels, as the increased BAL levels of these potential mitogens were not reduced with CGZ treatment [95]. It would be of interest to measure endogenous factors that could contribute to ASM proliferation in this setting.

### 6.4. Regulation of Airway Hyperresponsiveness

Studies demonstrating the inhibitory effects of PPAR*γ* ligands on AHR are consistent with the numerous *in vitro* findings suggesting a role for PPAR*γ* ligands in the regulation of ASM function in asthma (Table 1). The development of AHR to cholinergic agonists subsequent to *in utero* nicotine exposure or *in vivo* allergen exposure can be alleviated by chronic treatment with PPAR*γ* ligands, measured either indirectly using Penh [25, 27, 94, 95] or by assessing changes in airway resistance [47, 48, 97, 98].

A recent study has reported that coadministration of RGZ prevented the changes in lung function in rat offspring induced by perinatal nicotine exposure. Inhibition of the development of AHR as measured *in vivo* and in isolated tracheal preparations was attributed to the ability of RGZ to decrease the lipofibroblast-to-fibroblast transdifferentiation induced by nicotine, minimizing the increased expression and function of mesenchymal markers of contractility [48].

Chronic allergen studies demonstrating PPAR*γ* ligand efficacy suggest a PPAR*γ*-dependent mechanism in opposing ovalbumin-induced AHR, since the inhibitory effects could be mimicked by transient overexpression of PPAR*γ* via adenoviral delivery or prevented by co-treatment with GW9662 [25, 27, 47, 93, 94, 97, 98]. It would be reasonable to attribute this reduction in AHR to the inhibition of inflammation and airway remodeling mediated by the PPAR*γ* ligands used. However, RGZ also reduced AHR measured by invasive plethysmography in OVA-challenged C57Bl/6 mice without detectable effects on markers of inflammation or remodeling [98]. This result suggests that it is possible that PPAR*γ* ligands may also exert a direct effect on ASM contractile function *in vivo*.

In this context, it is notable that chronic treatment with PPAR*γ* ligands may not only inhibit the development of AHR, but also protect airway dilator responses. In a guinea pig model of *in vivo *homologous desensitization to salbutamol, chronic treatment with RGZ mitigated AHR to carbachol, preserved salbutamol relaxant activity, and partially restored *β*_2_-adrenoceptor binding sites in tracheal tissues *ex vivo* [105]. The potential for PPAR*γ* ligands to maintain dilator sensitivity and reverse *β*_2_-adrenoceptor desensitization is of particular interest since GCS can prevent cytokine-induced desensitization [106], but cannot restore sensitivity once tolerance to *β*_2_-adrenoceptor agonists has developed [107].

## 7. Potential Clinical Benefit of PPAR*γ* Ligands in Asthma

Although several members of the glitazone class of drugs have been used for type 2 diabetes, PGZ is the only PPAR*γ* agonist in current clinical use for this condition, with its potential as a treatment to reduce inflammation in rheumatoid arthritis also being assessed [108]. TGZ was the first glitazone to be marketed for diabetes, but was withdrawn because of serious hepatotoxicity in some patients [109], while RGZ has also recently been withdrawn because of potential cardiovascular risks [110].

There is currently only limited data on the efficacy of glitazones in the treatment of respiratory diseases. Further studies characterizing the effects of PPAR*γ* ligands on lung development as well as nicotine-induced changes in lung function are required to determine whether these agents may provide a new therapeutic approach to minimize, or even reverse, the adverse impacts of maternal smoking that contribute to the development of paediatric asthma.

An isolated report described the effects of PGZ in two case subjects with both diabetes and established asthma [111]. One patient reported reduced wheezing when taking PGZ in addition to his asthma preventer medication, with deterioration of symptoms when PGZ was discontinued. In another, concurrent treatment with the sulfonylurea glibenclamide and PGZ effectively reduced the patient's blood glucose levels and improved pulmonary function test results, increasing both forced vital capacity and force expiratory volume in one second (FEV1).

More recently, a small single-centre trial has been conducted, assessing RGZ in a double-blind, randomised, placebo-controlled, two-period crossover study in the inhaled allergen challenge model of asthma [112]. 32 steroid naïve subjects completed the study, receiving RGZ (4 mg) and placebo twice daily for 28 days in random order. The late asthmatic reaction (LAR) change from postsaline FEV1 from 4–10 hrs after allergen on day 28 was attenuated by 15% compared to the response during placebo-treatment, suggesting an inhibitory effect of RGZ on activated eosinophil recruitment. This reduction was accompanied by trends in several other markers of efficacy and anti-inflammatory activity (e.g., IL-4, IL-6, IL-13). In light of these modest changes, the authors suggested that PPAR*γ* agonist monotherapy is unlikely to represent a clinically useful intervention, at least in the context of relatively mild asthma.

More positive results were obtained in another recently completed exploratory clinical trial, which compared the effects of oral RGZ (8 mg) with inhaled beclometasone in a group of forty-six smokers with asthma, a group that is generally unresponsive to conventional GCS treatment [113]. In measurements taken after two and four weeks, RGZ did not significantly reduce asthma symptoms as determined by the Asthma Control Questionnaire (ACQ) scores and only produced a borderline reduction in sputum IL-8 levels compared to beclometasone-treated patients [113]. However, the patients receiving RGZ experienced significant improvements in FEV1 and forced expiratory flow over beclometasone-treated patients, which may reflect an effect of RGZ to reduce small airway obstruction.

These promising findings support the assessment of the effectiveness of long-term treatment of RGZ in a larger treatment group. The use of substantially higher oral doses may not be associated with a positive benefit/risk profile in asthma since PPAR*γ* agonists are associated with dose-related adverse effects such as weight gain (probably secondary to fluid retention). This suggests that a preferable alternative strategy would be to assess responses to both acute and chronic inhalation of PPAR*γ* agonists. This route of administration would potentially minimize the reported adverse cardiovascular effects that have limited the systemic use of RGZ in diabetes [110]. In addition, it would achieve the higher local airway concentrations that may be required to exert direct effects on ASM contractile function to elicit acute bronchodilation as reported in mouse trachea, and chronic effects to regulate airway inflammation, remodeling, and the development of AHR.

## 8. Summary 

**Figure 2:**
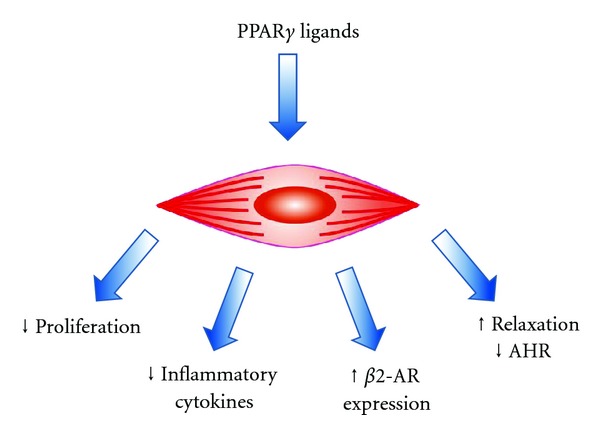
PPAR*γ* ligands regulate noncontractile and contractile functions of airway smooth muscle.

An accumulating body of evidence supports the use of PPAR*γ* ligands for the targeting of PPAR*γ* receptors and other PPAR*γ*-independent mechanisms in ASM for the treatment of inflammatory lung diseases (Figure 2) [9, 41, 114]. *In vitro* treatment inhibits proliferation of human ASM via PPAR*γ* [18, 55] and also inhibits cytokine release from these cells [55, 62, 63]. Chronic *in vivo* treatment inhibits the development of nicotine-induced AHR in rat airways [48] as well as OVA-induced increases in ASM mass in mouse airways [95], part of a suite of actions involving inhibition of airway inflammation, remodeling and the development of AHR. PPAR*γ* ligands may also protect dilator responses since they can preserve *β*_2_-adrenoceptor expression and function in a guinea pig model of homologous desensitization to albuterol [105]. The potential for direct bronchodilator actions is supported by the demonstration of acute PPAR*γ*-independent relaxation in mouse trachea [91]. Although clinical trial results are limited, evidence of improved lung function in a difficult-to-treat cohort of smokers with asthma [113] supports further investigation of the potential for PPAR*γ* agonists to target ASM proliferative, inflammatory and contractile functions and their contributions to impaired dilator responses and the consequences of AHR in asthma.

## Figures and Tables

**Table 1:** Effects of PPAR*γ* ligands in mouse OVA models of allergic airways disease.

Strain	Ligand	Inflammation	Remodeling	AHR	Mechanism	References
Balb/C	CGZ	↓ IL-4, IL-5, IL-6, IL-13 ↓ eosinophils	↓ mucus ↓ collagen ↓ wall thickness	↓	↓ GATA-3	[25, 94]
Balb/C	CGZ	↓ IFN*γ*, IL-2, IL-4 ↓ eosinophils	↓ mucus	N.D.		[100]
Balb/C	CGZ	↓ eosinophils	↓ ASM thickness	↓		[95]
Balb/C	CGZ RGZ	↓ eosinophils		N.D.	↑ IL-10	[99]
Balb/C	RGZ PGZ	↓ IL-4, IL-5, IL-13, ECP ↓ eosinophils		↓	↑ PTEN ↑ IL-10	[27, 47]
Balb/C	GI 262570	↓ eosinophils		N.D.		[92]

C57Bl/6	RGZ PGZ	↓ IL-4, IL-5, IL-13, ↓ VEGF, eotaxin, RANTES ↓ eosinophils		↓	↑ IL-17 via NF*κ*B	[93, 97]
C57Bl/6	RGZ	⇔ eosinophils	⇔ mucus ⇔ wall thickness	↓		[98]

ASM: airway smooth muscle; CGZ: ciglitazone; ECP: eosinophil cationic protein; IL: interleukin; N.D.: not determined; PGZ: pioglitazone; PTEN: phosphatase and tensin homologue deleted on chromosome ten; RANTES: regulated upon activation, normal T-cell expressed, and secreted; RGZ: rosiglitazone; VEGF: vascular endothelial growth factor.

## Abbreviations

15dPGJ_2_: 15-Deoxy-^Δ12,14^-prostaglandin-J_2_
ACQ: Asthma control questionnaire
AdPPAR*γ*: Adenoviral PPAR*γ*
AF-1: Activator function-1
AHR: Airway hyperresponsiveness
AMPK: Adenosine monophosphate-activated protein kinase
AP-1: Activator protein-1
AR: Adrenoceptor
ASM: Airway smoothmuscle
*α*SMA: *α* smooth muscle actin
BAL: Bronchoalveolar lavage
C/EBP: CAAT/enhancerbinding protein
CDDO: 2-Cyano-3,12-dioxoolean-1,9-dien-28-oic acid
CGZ: Ciglitazone
DBD: DNA binding domain
ECM: Extracellular matrix
ECP: Eosinophil cationic protein
FFA: Free fatty acid
FGF2: Fibroblast growth factor
GCS: Glucocorticoid
G-CSF: Granulocyte-colony stimulating factor
GM-CSF: Granulocyte/macrophage-colony-stimulating factor
GR: Glucocorticoid receptor
HETE: Hydroxyeicosatetraenoic acid
HODE: Hydroxyoctadecadienoic acid
IFN*γ*: Interferon*γ*
IB*κ*: NF*κ*B inhibitory cofactor
IKK: I*κ*B kinase
IL-: Interleukin
i.p.: Intraperitoneal
LBD: Ligand binding domain
MAPK: Mitogen-associated protein kinase
MCh: Methacholine
MMP: Matrix metalloproteinases
NF*κ*B: Nuclear factor *κ*B
NHR: Nuclear hormone receptor
OVA: Ovalbumin
Penh: Enhanced pause
PG: Prostaglandin
PGZ: Pioglitazone
PI3K: Phosphoinositide-3-kinase
PPAR*γ*: Peroxisome proliferator activated receptor
PPRE: PPAR response element
PTEN: Phosphatase and tensin homologue deleted on chromosome ten
RANTES: Regulated on activation, normal T cells expressed and secreted
RGZ: Rosiglitazone
RXR: Retinoid X receptor
SERCA: Sarco/endoplasmic reticulum Ca^2+^ ATPase
STAT: Signal transducers and activators of transcription
SUMO: Small Ubiquitin-like Modifier
TGF*β*: Transforming growth factor *β*
TGZ: Troglitazone
TNF*α*: Tumour necrosis factor *α*
VEGF: Vascular endothelial growth factor.
